# Properties of Cracking Patterns of Multi-Walled Carbon Nanotube-Reinforced Cement Matrix

**DOI:** 10.3390/ma12182942

**Published:** 2019-09-11

**Authors:** Maciej Szeląg

**Affiliations:** Faculty of Civil Engineering and Architecture, Lublin University of Technology, 40 Nadbystrzycka Str., 20-618 Lublin, Poland; maciej.szelag@pollub.pl; Tel.: +48-81-538-4428

**Keywords:** cement paste, cement matrix, cement, MWCNT, cracking patterns, elevated temperature, image analysis

## Abstract

The research presented in this paper presents a quantitative analysis of cracking patterns on the surface of cement paste, which has been modified by the addition of the multi-wall carbon nanotubes (MWCNTs). The cracking patterns analyzed were created as a result of increased temperature load. MWCNTs were used as an aqueous dispersion in the presence of a surfactant, sodium dodecyl sulfate (SDS). Four series of the cement paste were tested, and the samples differed in the water/cement (w/c) ratio, cement class, and the presence of MWCNTs. Image analysis tools were used to quantify the cracking patterns and it was proposed to measure parameters, such as the average cluster area, average cluster perimeter, average crack width, and crack density. In order to facilitate the image analysis process, the sample surface was subjected to preparation and using statistical analysis tools it was assessed whether the method of surface preparation affects the way the sample is cracked. The paper also presents the analysis of the relationships that occur between parameters describing the cracking patterns, and also with the physico-mechanical properties of the cement pastes. It was attempted to explain the dependencies using elements of fractal theory and the theory of dispersion systems.

## 1. Introduction

Cementitious composites embedded in a real building structure are subjected to constant stresses and deformations due to changes in environmental parameters, e.g., changes in air humidity, air temperature, sunlight, etc. The effect of these deformations is the development of cracks in the material that arise in its weakest place [1,2]. As a result of propagation, these cracks merge and form a characteristic crack image on the surface of a cementitious element, known as the cracking pattern. Depending on the literature source and cracking factor, the cracking patterns are also referred to as the cluster cracks, thermal cracks, or mapcracking [3,4,5]. The cracking patterns described using the quantitative parameters, such as the crack density or crack width, determine the further durability and strength of a cementitious material because the progressive development of cracks is a simple method of the material’s destruction.

In order to be able to quantitatively describe the cracking patterns, the concept of the cluster [3,4,6] was introduced. It is defined as the area on the surface of a material which, on each side, is limited by a crack or the edge of a sample. Clusters have a fractal character and are observable in both the micro- and macrostructure of the material. In order to describe the formation process of the cluster structures, the theory of dispersion systems is used because it is assumed that the final properties and structure of the material are the result of transformation and physical interactions occurring in the binder-water dispersion system, i.e., when a cement paste is still in the liquid phase. The properties of such a system depend on many factors, such as the cement grain size, w/c ratio, and the presence of pozzolanic additives or inert fillers [7,8,9]. The final properties of the cement matrix are largely dependent on the spatial configuration of cement grains. They, when hydrated, form a mutually tied, rigid structure composed of hydration products that are able to transfer external loads. In this structure, air voids are also present, which are defects and the places where the cracks begin to develop [10,11]. These cracks propagate as a result of increasing material deformations resulting from external influences and eventually create a cracking pattern that can be observed in both micro- and macro-scale.

Nanotechnology is the future of every field of technical sciences, which is why research on the material modification at the nanoscale is also carried out in the context of cement matrix modification. Nano-modification of a cement matrix usually involves two directions, i.e., introducing nanoparticles into the cement paste, which can seal the structure or possess pozzolanic properties, which are the main, but not the only, function in the reaction with cement components to form the CSH phase [12,13,14,15]. Additionally, the introduction of dispersed nano-reinforcement mechanically bonds the structure of a cement matrix, causing the phenomenon of crack bridging [16,17]. In this context, in many countries of the world, research is ongoing on the use of carbon nanotubes as the dispersed nano-reinforcement of a cement matrix [18,19,20,21,22]. The results of research to date indicate that the addition of CNTs may improve the mechanical properties of a cement matrix, increase the crack resistance, increase the local rigidity of the CSH phase [16,17,23], or reduce the porosity [24].

Carbon nanotubes (CNTs) can occur as single- (SWCNTs), double- (DWCNTs), and multi-wall (MWCNTs) structures [25]. They have unique mechanical parameters (the Young’s modulus about 2 TPa, and tensile strength of about 50 GPa), and very good thermal and electrical conductivity. Depending on the manufacturer, CNTs with a diameter of 1–100 nm and a length of 10 nm–10^−2^ m can be found on the market [26,27,28]. The process of obtaining them is energy-consuming and requires specialized equipment [26,29], which means that this material is still expensive, but due to its properties, it is the future of many engineering fields.

The use of CNTs in concrete technology is very problematic due to its tendency to form strongly bonded agglomerates, which causes them to be entangled in large aggregates. This is due to the fact that CNTs have a large specific surface area and a large slenderness. Using them in such a form as an addition to the cement matrix does not cause any beneficial effects, but generates only unnecessary costs. To prevent this (i.e., “untangle”) they should be used in the presence of a surfactant, which, by absorbing on the surface of CNTs, causes them to repel each other. In the context of the use of CNTs for cement pastes, their dispersion is usually made in water with a surfactant, which is then added to the cement and mixed. Research is continuing to find a surfactant that is as neutral as possible to the cement paste. To this end, the CNTs’ dispersions have already been made in the presence of, among others, H_2_SO_4_, HNO_3_ [24,30], isopropanol [23], sodium dodecyl sulfate (SDS) [31,32,33,34,35,36], sodium dodecyl benzene sulfonate (NaDDBS) [37], and di-methyl acetamide (DMAc) [31]. By using the above surfactants, a stable dispersion of CNTs is obtained, even over a period of several months.

Quantitative analysis of cracking patterns is difficult for methodological reasons, therefore, there are few such analyzes in the literature. However, numerical analyzes were carried out regarding the process of developing the cracking patterns in a cement matrix [2,38,39]. The most commonly used parameter for the quantitative description of cracking patterns is the crack density [40,41,42], which was defined and used for the first time by Mobasher et al. [43]. Kim et al. [44], as a result of the conducted analyzes, pointed out that the phenomenon of spalling occurring in concrete exposed to fire temperatures is mainly caused by the formation of a crack network, which significantly weakens the material structure. Weng et al. [45] observed cracking patterns resulting from shrinkage of the cement paste, which was modified by various types of fibers. The total area of cracks was smaller compared to the classical cement paste. In [46] it was pointed out that the cracking pattern’s characteristics on the cement matrix surface depends on such parameters as drying rate, the sample thickness, and the crack depth. The author of this work in his earlier studies conducted a quantitative analysis of the cracking patterns on the surface of a cement paste modified with metakaolinite [47], microsilica [48], and polypropylene fibers [49].

The purpose of the research presented below was to determine how the addition of the dispersed nano-reinforcement in the form of MWCNTs affects the quantitative characteristics of the cracking patterns on the surface of a cement matrix. A total of four series of a cement paste were tested, which differed in the class of cement used and the presence of MWCNTs. For quantitative analysis of the cracking patterns, the computer image analysis and four parameters were used, such as the average cluster area, the average cluster perimeter, the average crack width, and the crack density. To facilitate the image analysis process, the samples’ surfaces were prepared by applying a thin film of white color. Using the statistical analysis tools, the article assesses whether the preparation method used affects the cracking process of the surface of the cement paste. This work is a continuation of the research described in [50], where the physico-mechanical properties of cement paste with the addition of MWCNTs were analyzed in detail.

## 2. Materials and Methods

### 2.1. Components Used and Mixtures

The binder used to make the cement matrix was ordinary Portland cement (OPC) of two different classes—CEM I 42.5R and CEM I 52.5 (Cemex, Chelm, Poland). Both cements were very similar in chemical composition; content of SiO_2_—20.18% and 20.19% (respectively for CEM I 42.5R and CEM I 52.5R); CaO—64.79% and 64.76%; Al_2_O_3_—4.38% and 4.33%; Fe_2_O_3_—3.39% and 3.30%; MgO—1.17% in both; SO_3_ —2.91% and 3.16%; Cl—0.083% and 0.078%; Na_2_O—0.26% in both; K_2_O—0.49% and 0.48%.

Using Bogue’s formulas [51], the percentage content of the main cement phases was calculated, i.e., alite (C_3_S)—63.41% and 62.97%, belite (C_2_S)—8.92% and 9.28%, tricalcium aluminate (C_3_A)—5.88% and 5.90%, and tetra calcium aluminate (C_4_AF)—10.31% and 10.03%. Like the chemical composition, the phase composition is very similar. However, the cements used differ from each other in the degree of grain fragmentation (fineness), because the CEM I 42.5R has the Blaine’s specific surface area of 4010 cm^2^/g, while the CEM I 52.5R–4596 cm^2^/g. The difference in the surface area is 14.6%. With almost identical chemical and mineral composition, the difference in grain size affects, among others on the rate of hydration of cement grains, or the number of connections between them. This has a large impact on the final properties of a hardened cement matrix.

**Figure 1:**
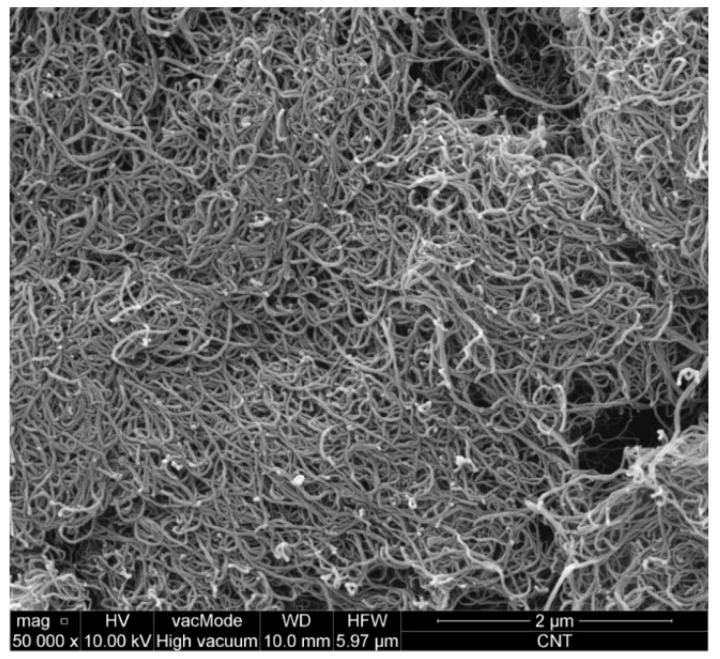
SEM image of MWCNTs used at 50,000× magnification; a visible tendency to agglomerate individual nanotubes into larger aggregates.

The cement matrix has been reinforced with the multi-wall carbon nanotubes (MWCNTs) of the NC 7000 type, manufactured by the Nanocyl^TM^ company (Sembreville, Belgium). According to the technical card, they are characterized by an average length of 1.5 μm, average diameter—9.5 nm, specific surface area—250–300 m^2^/g, Young’s modulus—1 TPa, tensile strength—60 GPa, and carbon purity—90%. Figure 1 shows image of MWCNTs used in the study, obtained from a scanning electron microscope (SEM, Quanta Feg 250, FEI, Hillsboro, OR, USA).

Due to the very large specific surface area, MWCNTs tend to agglomerate forming centers of nanotubes coiled together. Applying them in this form to a cement matrix has no effect, so they should be evenly distributed. For this purpose, MWCNTs were applied to the cement matrix in the form of an aqueous solution in which the surfactant—sodium dodecyl sulfate (SDS—C_12_H_25_OSO_2_ONa) was used (Merck Milipore, Billerica, MA, USA). It is a white powder with a bulk density ranging from 0.49 to 0.56 g/cm^3^, specific density—1.1 g/cm^3^, and pH—6–9.

**Figure 2:**
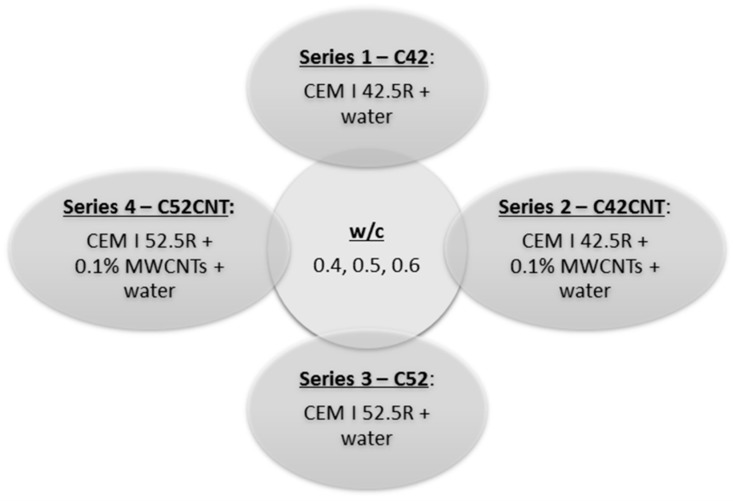
Designations and composition of the series.

As part of the tests conducted, four series of cement pastes were made, which consisted of twelve different recipes. It is schematically shown in Figure 2. Within each series, samples were made with three water/cement (w/c) ratios—0.4, 0.5, and 0.6. In the series in which MWCNTs were used, their content was assumed at the level of 0.1% in relation to the mass of cement.

### 2.2. Procedure of the Mixture Preparation

Cement paste was created in the same way as in [50], i.e.,:

- the cement and water mixture was mechanically mixed until it obtained a uniform consistency,
- MWCNTs was used as the aqueous solution with surfactant (SDS); then this aqueous solution was mechanically mixed with cement,
- the MWCNTs/SDS weight ratio was 1:5,
- an aqueous MWCNTs-SDS solution was sonicated for a period of 30 min; sonication took place in a glass jar, which was placed in a bucket of cold water to remove excess heat resulting from ultrasonic mixing; the amount of the solution produced at one time was sufficient to produce six samples of the cement paste,
- for the sonication, the UP400S ultrasonic homogenizer of the HIELSCHER Ultrasound Technology company (Teltow, Germany) was used, it was equipped with the H22 horn sonotrode with a tip diameter of 22 mm, maximum amplitude—100 μm, sound power density—85 W/cm^2^, operating frequency—24 kHz; sonication took place in the continuous mode of the device operation, with maximum power, which in connection with the H22 sonotrode giving 300 W of power,
- test samples were made as 40 × 40 × 160 mm beams, which is in line with the requirements for testing cement pastes and mortars; the mix was laid in molds in two layers, successively compacted, using a standardized shaker, in accordance with the EN 196-1 [52], and
- samples were demolded 24 h after forming; maturation took place in dry-air conditions (average air temperature—22 °C, average relative air humidity—50%; maturation period was equal to 28 days).

### 2.3. Cracking Patterns Formation Process

The cracking patterns being the subject of the analysis were created as a result of loading the material with an increased temperature. The thermal load process consisted of three phases:

- Phase I—preheating the furnace to 250 °C,
- Phase II—placing the samples in the furnace and heating them at the above temperature (250 °C) for a period of 4 h, and
- Phase III—removing samples from the furnace and cooling them as a result of a natural decrease in the samples temperature under laboratory conditions (average air temperature—20 °C, average relative air humidity—50%); the samples reached ambient temperature within two hours of being removed from the furnace.

The application of the thermal load procedure causes the cement paste to be subjected to the thermal shock. In the case of a cement matrix, which contains some free water in its volume, the application of a thermal load on the principle of the thermal shock causes much more damage to the material structure than it would be in the case with gradual heating. The main destructive factor is the effect of saturated steam pressure, which is more intensified in the case of the thermal shock. Not without significance is the occurrence of a large temperature gradient between the inside of the sample and its external surface. This leads to the formation of stresses associated with the thermal deformation of the material.

**Figure 3:**
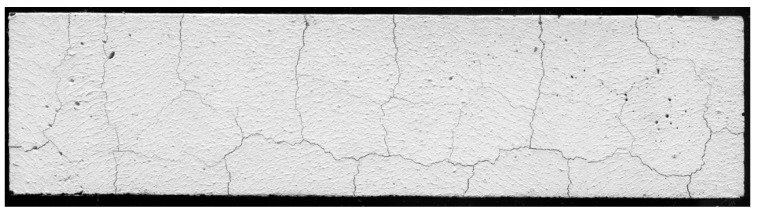
Cracking pattern on the surface of the cement matrix with MWCNTs (C52CNT) with w/c = 0.4.

The saturated steam pressure causes the local tensile strength of the material to be exceeded, which results in cracks on the surface and in a volume of the cement matrix. In addition, this phenomenon is compounded by volume changes of the paste, i.e., swelling due to the heating (the cement matrix, like most physical bodies, is subjected to the thermal expansion) and shrinkage in the cooling phase. The resultant of these interactions is the formation of a characteristic cracking pattern (an example is shown in Figure 3) on the surface of a cementitious material. Previous studies [53] have proved that the cement matrix is chemically stable in the temperature range up to 250 °C (up to this temperature occurs, among others breakdown of the brownmillerite, Ettryngite, or gypsum phases), and the main structural damage is caused by physical changes occurring in the matrix volume.

### 2.4. Basic Physico-Mechanical Properties of the Cement Matrix

Szelag [50] describes in detail the research methodology and results of physico-mechanical properties of the cement pastes tested. Table 1 summarizes the values of the most important parameters, i.e., compressive strength (*f_c_*), tensile strength (*f_cf_*), apparent density (*D*), and shrinkage after the maturation period (*S*_28_). The (*R*) index represents the values obtained on the reference samples (after 28 days of maturation), the (*T*) index represents the values determined for samples that have been subjected to the thermal load.

### 2.5. Quantitative Analysis of the Cracking Patterns—Image Analysis

Quantitative analysis of the cracking patterns was carried out using computer image analysis techniques. For this purpose, the ImageJ v. 1.51j8 software (National Institute of Health, Bethesda, Rockville, MD, USA) was used, and the flat image of the cracked surface of the cement paste sample was analyzed. The image was obtained by scanning the surface on an optical scanner. In order to obtain a very detailed image of the sample surface, the scanning was carried out in a very high 2400 DPI resolution. Cracks are identified at their width of about 2–3 pixels, which at this scanning resolution gives a width of about 0.021 mm. Thus, by scanning the sample in such a high resolution, cracks can be identified that are not visible to the naked eye which, in the case of scanning without any additional magnifying optics, should be considered as a very good result.

To quantify the cracking patterns, the following parameters were used:

- $\bar{A}$—the average cluster area (mm^2^),
- $\bar{L}$—the average cluster perimeter (mm),
- $\bar{I}$—the average crack width (mm),
- *CD*—the crack density (m^−1^).

In the case of $\bar{A}$ and $\bar{L}$, each cluster present on the sample surface was measured. For this purpose, the “analyze particles” module was used. The “plot profile” module was used to measure $\bar{I}$. The crack width was measured along a line parallel to the longitudinal axis of the sample, located halfway across the sample width. In the case of *CD*, the measurement for each sample was made along 3 lines parallel to the longitudinal axis of the specimen. The measuring lines were arranged so that they divided the sample surface into four equal parts. The values of all parameters describing the cracking patterns are the average of the measurements taken on four samples. Detailed procedures for measuring the $\bar{A}$, $\bar{L}$, $\bar{I}$, and *CD* values using ImageJ v.1.51j8 software are described in Appendix A.

**Figure 4:**
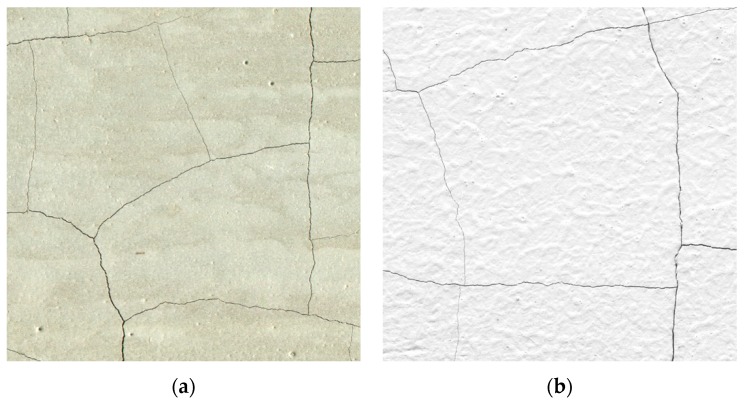
Scanned fragment of the cement paste surface: (**a**) before applying the acrylic film; (**b**) after applying the acrylic film.

The process of image analysis of the unmodified sample surface causes many difficulties in properly separating the cracks from the remaining surface of the sample (cracks are dark gray or black, the remaining areas on the sample are gray). Due to the unsatisfactory contrast of cracks in relation to the cluster surface, the graphical processing and the image analysis become very time and labor consuming. Additionally, there may be a risk that elements of the phase tested may be mistakenly removed in the course of image processing, which results in an incorrect result of the analysis. Thus, the author decided to prepare the sample in order to achieve greater contrast between the cracks and the clusters even before scanning. In addition, the goal was to use such means that the surface of the element operating in a real construction could be prepared in such a way, not only in the laboratory conditions. Thus, it was decided to use a thin acrylic film (Figure 4) in white color on the tested surface of a sample. The film was made using a painting method, by a roller. After the film had dried, the white surface of the clusters was in great contrast compared to cracks formed by the temperature load. This significantly improved the process of further processing of the scans.

In the case of applying the thin film on the entire scanned surface of the sample, it is important to examine whether, as a result of the increased temperature, the film cracked independently of the sample, whether the cracks on the film reflect the crack network on the actual surface of the sample. For this purpose, additional samples of one of the series (C42) were made and subjected to the $\bar{A}$ and $\bar{L}$ measurement procedure, but before the thermal loading the samples were not subjected to the surface preparation. The result was an analysis of the actual sample surface. Then the results were compared with the results obtained on samples of the same series but subjected to the preparation process.

## 3. Results

**Figure 5:**
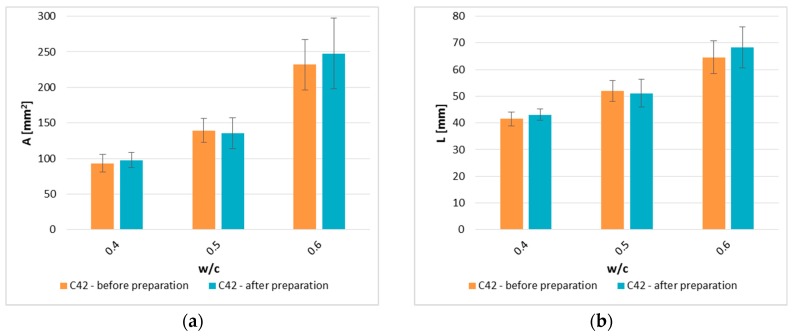
Comparison of the image analysis results between samples of the C42 series that have not and have been subjected to the surface preparation: (**a**) $\bar{A}$—the cluster average area; (**b**) $\bar{L}$ —the cluster average perimeter.

Figure 5 shows the $\bar{A}$ and $\bar{L}$ results for samples of the C42 series in two variants, i.e., samples without a thin white acrylic film and samples with the film. At the furnace temperature (250 °C), the film has not undergone any damage that could occur, e.g., in the form or discoloration. The use of a white film on the surface of the cement paste reduced the time-consumption of the measuring process.

**Figure 6:**
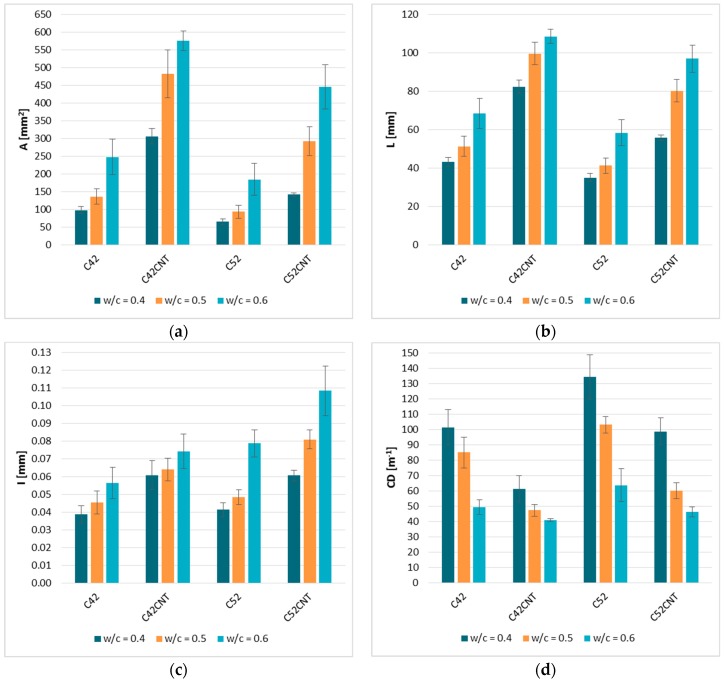
The results of the quantitative characteristics of the cracking patterns: (**a**) $\bar{A}$—the cluster average area; (**b**) $\bar{L}$ —the cluster average perimeter; (**c**) $\bar{I}$ —the average crack width; (**d**) CD —the crack density. Error bars represent the standard deviations.

The results of measurements from the image analysis for all series of cement paste are presented in Figure 6. When assessing the standard deviation values for individual parameters, there are no grounds to undermine the reliability and repeatability of the results. The obtained results indicate that the geometrical characteristics of the cracking patterns depend on the technological variables in the cement paste production process, i.e., the class of cement used (in this case the degree of fragmentation of cement grains), the presence of dispersed reinforcement in the form of MWCNTs, or the w/c ratio (the greatest impact).

## 4. Discussion

### 4.1. Assessment of the Impact of the Surface Preparation Method on the Correctness of the Results Obtained from the Image Analysis

The average $\bar{A}$ and $\bar{L}$ values for samples before and after preparation are shown in Figure 5. $\bar{A}$ for samples after preparation differs from the results obtained on samples which surface was not modified by 4.5% for w/c = 0.4, 2.7% for w/c = 0.5, and 6.2% for w/c = 0.6. Correspondingly, $\bar{L}$ results obtained differ by 3.7%, 1.7%, and 5.4%. It can be concluded that the use of the thin film does not affect the results obtained because in four out of six cases the difference between the parameters is less than 5%. However, the differences in results for samples with w/c = 0.6 slightly exceed this threshold. Statistical analysis tools were used to confirm the thesis that the thin film applied on the sample surface did not affect the results obtained of parameters describing the cracking patterns.

In order to select the appropriate statistical test to show whether the differences obtained between the measurements are statistically significant, the distribution similarity of the $\bar{A}$ and $\bar{L}$ results in the normal distribution being checked by conducting the Shapiro–Wilk test. The null hypothesis is tested stating that the $\bar{A}$ and $\bar{L}$ distribution is close to the normal distribution. It follows that a significant test result will show that the distribution of the variables observed is not similar to the normal distribution—the alternative hypothesis. The test was conducted in Microsoft Excel using the official *Analysis ToolPak* package and the free *Real Statistics* add-on [54]. The significance level α = 0.05 was assumed. The test consists in calculating the *W* statistics, followed by the *p*-value. If the *p*-value *≤ α* then the rejection of the null hypothesis about the similarity of the distribution to the normal distribution occurs. The test results are presented in Table 2.

The population of results subjected to the statistical analysis consisted of all clusters that formed on the surface of the cement matrix (four samples for each w/c, both in the group of samples subjected to and not subjected to the surface preparation). The number of individual results slightly differed between the groups compared. In the case of comparative statistical tests, the result is more objective if the compared groups have the same or similar numbers. Thus, using the *Sampling* function available in the *Analysis Tool Pak*, samples from the population of results were randomly selected so that the number of groups compared in subsequent analysis was the same.

Analyzing the results of the Shapiro-Wilk test, it was noticed that in 11 out of 12 groups of results the *p*-value *≤ α* was met. The null hypothesis was rejected and the alternative hypothesis was considered true—the distribution of individual groups of results does not show similarity to the normal distribution.

In order to check if there are statistically significant differences between the respective groups of results, one of the most popular nonparametric variability tests was used—the Mann-Whitney *U* test for independent samples (it does not require normality of the distribution, which is necessary in the case of e.g., the Student’s t test for independent samples). The null hypothesis is tested saying that the samples come from one population—there are no statistically significant differences between the groups tested. The alternative hypothesis is that the samples come from different populations. As above, the Mann-Whitney *U* test was conducted in Microsoft Excel with the official *Analysis Tool Pak* add-on and the free *Real Statistics* add-on [54]. The significance level α = 0.05 was assumed. The test consists in ranking the results, then calculating the *U* statistics and then comparing them with the *U_crit_* statistics value. If *U ≤ U_crit_* then the null hypothesis is rejected. The test results are presented in Table 3.

Based on the results of the Mann-Whitney *U* test obtained for each of the compared groups, there is no reason to reject the null hypothesis, therefore it was accepted. Thus, in the case analyzed, it means that the thin white acrylic film applied can be used, because it does not significantly affect the correctness of the results obtained. The cracks formed on the film coincide with the cracks formed on the real surface of the sample—there are no statistically significant differences in the $\bar{A}$ and $\bar{L}$ results between the treated and untreated samples.

### 4.2. Characteristics of the Cracking Patterns

#### 4.2.1. $\bar{A}$—The Average Cluster Area

The $\bar{A}$ results for a particular series of cement pastes are presented in Figure 6a. An analysis of how the size of clusters is shaped can be made taking into account three basic variables, i.e., the w/c ratio, the class of cement used, and the presence of MWCNTs. The cluster area increases significantly with the increase in the w/c. Pastes with the w/c = 0.5 obtained higher $\bar{A}$ values by 39.1%, 57.5%, 43.3%, and 106.9% (for C42, C42CNT, C52, C52CNT, respectively) from a cement matrix with the w/c = 0.4. However, in the case of the w/c = 0.6 these differences were, respectively, 153.5%, 88.1%, 184.3%, and 215.0%. It is noteworthy that firstly the matrix made of CEM I 42.5R (C42 and C42CNT), i.e., cement with a larger specific surface area, were characterized by smaller relative increases in $\bar{A}$ compared to C52 and C52CNT samples; and secondly, the presence of MWCNTs in the cement matrix structure enhanced the relative increase of $\bar{A}$ compared to the samples without dispersed nano-reinforcement. In the extreme case, for the C52CNT series, samples with the w/c = 0.6 had $\bar{A}$ up to three times larger than the samples of the same series, but with the w/c = 0.4.

The degree of concentration of cement grains in a unit volume of the matrix is indirectly expressed by the w/c ratio. For example, the lower the w/c ratio, the greater the packaging degree of cement grains in the matrix. This affects the distance between individual cement grains and the grains hydration degree per unit of time. This ultimately translates into the amount and strength of local connections between hydrated cement particles [7]. Considering the fractal nature of the cracking patterns [55] and the fact that cracks appear and develop in the weakest places of the material, it is possible to translate the microstructure of the cement matrix into the image of cracks visible on the macro-scale on the sample surface, and probably also inside it. Cement grains flocculate immediately after combining with water, creating binding aggregates, which consist of a large number of grains [11]. Such aggregates are also of a fractal nature and occur at various levels of structural heterogeneity of the material, i.e., primary aggregates consisting of n-grains of cement form a cluster at a higher level of observation, etc., until there are observable clusters at the macro-scale. A significant feature of the binding clusters/aggregates is the fact that there is a separation surface between clusters on their periphery [4,6]. A crack forms between the separation surfaces of two adjacent clusters, which evolves as a result of stresses and deformations appearing in the material. The stresses and deformations are caused by external interactions (e.g., increased temperature). These cracks at a higher level of the structure create a characteristic cracking pattern that is the subject of the analysis. As the w/c ratio increases, the packing degree of cement grains in the cement matrix volume decreases, which causes a decrease in its bulk density, which was observed for each series tested (Table 1). The author has already proved in his earlier considerations [48] that along with a decrease in the material density, the clusters are formed of ever larger sizes and increasingly larger distances between the separation surfaces between clusters.

The use of cement with a greater degree of fragmentation affected the $\bar{A}$ reduction. The relative influence of the class of cement used is the greater the smaller the w/c is, because the samples of the C52 and C52CNT series with the w/c = 0.4 had $\bar{A}$ values lower by 33.7% and 53.8%, respectively, than the samples of the C42 and C42CNT series. The same difference for samples with the w/c = 0.5 was 31.7% and 39.3%, and for the w/c = 0.6—25.7% and 22.6%. It has not been observed that the presence of MWCNTs clearly affected the $\bar{A}$ value in the context of the cement class used.

Flocculation of cement grains in the aqueous suspension is caused by capillary forces between them. The value of these capillary forces depend on the size of the cement particles and is greater the larger their size is [6]. The result is the possibility of nucleation of larger binding aggregates due to the presence of larger cement grains or due to the possibility of fixation of a binding aggregate consisting of a larger number of cement grains.

The addition of MWCNTs caused a very large increase in the average cluster area. Samples of the C42CNT series had higher $\bar{A}$ values than the samples of the C42 series by 213.5%, 254.9%, and 132.5% (for the w/c = 0.4, 0.5, and 0.6, respectively). The same relative difference but between samples of the C52CNT and C52 series was 118.4%, 215.3%, and 142.0% respectively. In the case analyzed it is extremely difficult to determine how the MWCNTs addition itself influences the cracking patterns because the cement paste with the addition of MWCNTs had a much lower density than the reference cement matrix (Table 1). This difference in density is due to the fact that the use of MWCNTs as an aqueous dispersion in the presence of the surfactant (SDS) caused the cement paste to exhibit foaming properties during mixing. This phenomenon is further discussed in [50]. Thus, the above conclusions relate to the total impact of MWCNTs and SDS on the cracking patterns, where in this particular case the key effect of such a combination of dispersed reinforcement with a surfactant is a significant reduction in the bulk density of the material and, thus, an increase in the porosity of the cement matrix structure.

#### 4.2.2. $\bar{L}$—The Average Cluster Perimeter

Comparing the $\bar{A}$ (Figure 6a) and $\bar{L}$ (Figure 6b), a similarity of the occurring relationship was observed, i.e., an increase in the $\bar{L}$ value along with an increase in the w/c, the presence of MWCNTs causes the $\bar{L}$ to increase, and the use of more fragmented cement (CEM I 52.5 R) causes a decrease in the $\bar{L}$ value. The global correlation coefficient between the $\bar{A}$ and $\bar{L}$ parameters takes a very high value equal to 0.98. This proves the almost perfect correlation, which means that the mutual relationship between the surface and the perimeter of clusters is practically constant in every case. This is another proof that confirms the fractal nature of the cracking patterns—the geometric dependence of clusters is the same, regardless of their size.

**Figure 7:**
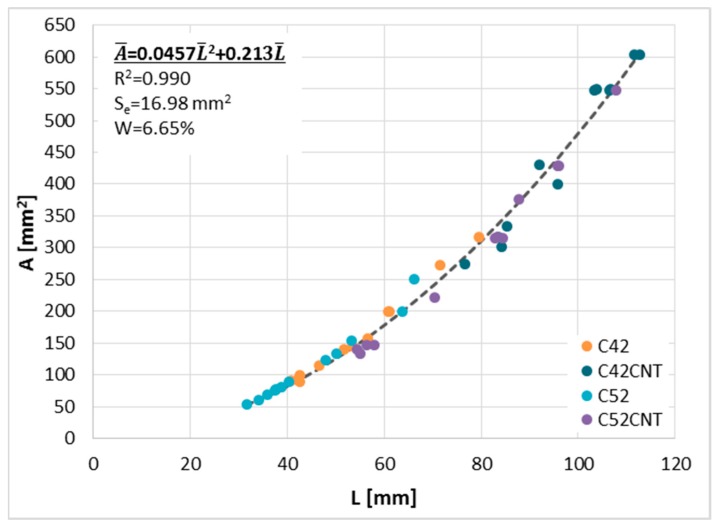
Global relationship—$\bar{A}\left(\bar{L}\right)$.

With this in mind, Figure 7 shows $\bar{A}$ as a function of $\bar{L}$ for all samples tested. The global nature of the relationship allowed the calculation of the functional curve equation with the help of which a very accurate estimation of $\bar{A}$ based on L can be performed. To this end, the least squares method (LSM) was used, and the quality of curve fitting to the empirical data was assessed by calculating three diagnostic statistics, i.e., determination coefficient (*R*^2^), standard deviation of estimation (*S_e_*), and coefficient of random variation (*W*) [56,57]. In order to ensure the mathematical correctness of the issue analyzed, it was assumed that the function will pass through the (0,0) coordinate and the equation is true for a set of positive real numbers.

The coefficient of determination takes a very high value (0.99), which indicates that the curve is almost perfectly matched to the data obtained from the measurements. The coefficient of random variation is equal to 6.65%, which means that the estimation error is very low. Due to the very strong relationship between $\bar{A}$ and $\bar{L}$, in further considerations the other parameters characterizing cracking patterns are dependent on one of these two parameters—$\bar{A}$.

#### 4.2.3. $\bar{I}$—The Average Crack Width

The average crack width for individual cement paste series is shown in Figure 6c. Similarly to $\bar{A}$ and $\bar{L}$, the most visible relationship is the increase in the $\bar{I}$ value with the increase of the w/c ratio, regardless of the series. The $\bar{I}$ values for cement pastes with w/c = 0.5 were, on average, higher by 17.5%, 5.3%, 17.1%, and 33.1%—for the C42, C42CNT, C52, and C52CNT series, respectively—than the cement matrix with w/c = 0.4. The analogous relationship for samples with the w/c = 0.6 was 46.4%, 22.2%, 90.5%, and 78.4%, respectively. In the context of the w/c, the use of cement with a greater degree of fragmentation contributed to the relative growth of $\bar{I}$. However, taking into account the presence of MWCNTs, a relative decrease in $\bar{I}$ growth was observed, which may be caused by the bridging effect of the MWCNTs.

The crack width is a key parameter in assessing the durability and strength of a material. As the width of the cracks increases, the material becomes more and more susceptible to mechanical destruction, as well as becoming less and less resistant to the aggressive action of chemical agents, which can penetrate at a greater depth of the material. As noted earlier, as the w/c ratio increases, the content of cement grains in the finite volume is smaller, which results in an increase in their dispersion. This affects the increase in the distance between the grains and, finally, the increase in the distance between the centers of the binding aggregates. As a result of the thermal load applied, the deformations increase as the w/c increases because more water is able to evaporate from the sample.

Cement matrix made of more finely fragmented cement had a higher $\bar{I}$ values, which increased relatively with the increase of the w/c ratio. The cement paste of the C52 series had an $\bar{I}$ value on average higher by 7.0%, 6.6%, and 39.2% than the C42 series—for w/c = 0.4, 0.5, and 0.6, respectively. The analogous relationship between the C52CNT and C42CNT series took the values equal to 0.0%, 26.4%, and 46.0%. It is worth noting that the cement matrix with the w/c = 0.4, in which MWCNTs were used, had the same average crack width, regardless of the class of cement used. However, increasing the w/c ratio the relative differences in the $\bar{I}$ value also increases. From [11], it results that the tendency to flocculation is stronger when the cement grains are smaller and, thus, the resulting binding aggregates are more packed. This causes an increase in the distance between separation surfaces between clusters, which is identified with an increase in $\bar{I}$.

The use of MWCNTs increased the $\bar{I}$ value, and so the C42CNT samples were characterized by higher $\bar{I}$ values by 57.3%, 41.0%, and 31.3% on average than cement matrix without MWCNTs (C42)—for w/c = 0.4, 0.5, and 0.6, respectively. The same relationship but between the C52CNT and C52 series were equal to 47.1%, 67.3%, and 37.8%, respectively. In the case of pastes made with CEM I 42.5R, there is a clear relative decrease in the $\bar{I}$ value with increasing the w/c. In the case of samples made of CEM I 52.5 this relationship is no longer unambiguous.

With good dispersion [23,24], MWCNTs cause crack bridging, which has a positive effect on limiting the increase in crack width with increasing deformation in the material. In the case under analysis, this situation would probably also occur if it were not for the fact that the use of MWCNTs in the presence of SDS caused foaming of the cement matrix (significant reduction in a bulk density). A decrease in density and, thus, an increase in porosity and an increase in the degree of dispersion of cement particles in the material volume are the main factors causing an increase in $\bar{I}$.

**Figure 8:**
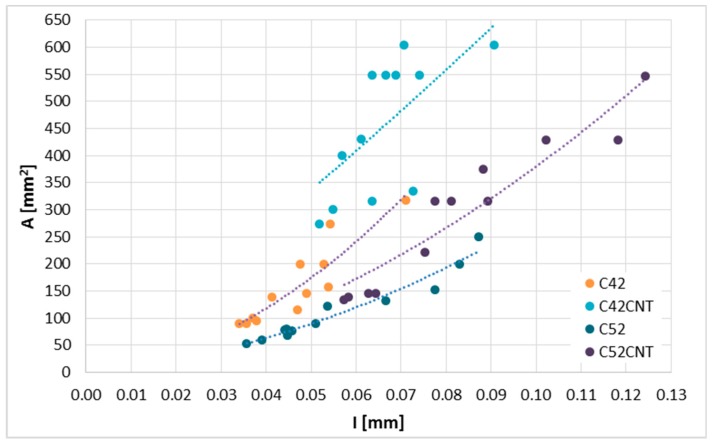
The $\bar{A}\left(\bar{I}\right)$ relationship divided into series.

Figure 8 shows the $\bar{A}$ value as a function of $\bar{I}$. As the global correlation coefficient between these two parameters is set to 0.66, it was decided to present this relationship with a division into the series. As in the case of $\bar{A}\left(\bar{L}\right)$, the functional curves serving the purpose of estimation were calculated using the LSM. The form of equations for each series, along with the values of diagnostic statistics are summarized in Table 4. As in the case of the $\bar{A}\left(\bar{L}\right)$, it was noted that the use of the second-degree polynomial gives the greatest degree of curve fit to the empirical data. To obtain the mathematical correctness of the relationship, the curves should pass through the (0,0) coordinate. The $\bar{A}\left(\bar{I}\right)$ relationship for all series except C42CNT is characterized by a very high correlation, i.e., ρ > 0.9. It is worth noting that despite a much lower degree of fit of the curve calculated for the C42CNT series, the relative estimation error takes a very similar value as for the C42 series. The best degree of curve fitting to the empirical data was obtained for samples made with CEM I 52.5R (C52 and C52 CNT)—*R*^2^ ≥ 0.9, and the *W* values are the smallest of all series. Thus, the estimation of $\bar{A}$ based on $\bar{I}$ for these two series is characterized by the highest accuracy.

#### 4.2.4. CD—The Crack Density

The crack density (Figure 6d) is a parameter that indirectly informs how much the material is cracked, because it tells about the number of cracks present on a unit of the material length. It was noticed that as the w/c increased, the *CD* value decreased, which indicates a smaller number of cracks. Thus, the cement matrix with the w/c = 0.5 had lower *CD* values on average by 16.2%, 22.6%, 23.3%, and 39.2% from pastes with the w/c = 0.4 (for C42, C42CNT, C52, and C52CNT, respectively). In the case of the w/c = 0.6, the above relationship took the following values—51.3%, 33.0%, 52.6%, and 53.2%. However, it should be remembered that due to the durability of the material and its resistance to environmental conditions, the width of the cracks is much more important. As mentioned earlier, along with the increase in the w/c, the width of the cracks increased, which, despite their smaller number, makes the material more susceptible to destruction. It was also confirmed by the results of the mechanical tests.

Analyzing the results in the context of the class of cement used, it was noticed that more fragmented cement (CEM I 52.5R) promotes the formation of fewer cracks. The *CD* value for the C52 series was 32.5%, 21.3%, and 29.0% higher than the C42 series (respectively for the w/c = 0.4, 0.5, and 0.6). Comparing the C52CNT and C42CNT series, the differences were 61.7%, 27.0%, and 13.0%, respectively.

The addition of MWCNTs caused a reduction in the number of cracks present in the material. The C42CNT cement paste reached *CD* values lower by 39.8%, 44.4%, and 17.2% than the C42 matrix (for the w/c = 0.4, 0.5, and 0.6). The above relationship for the C52CNT and C52 series has been set to 26.5%, 41.8%, and 27.5%, respectively. The results of the tests of mechanical features (Table 1) indicate that the occurrence of a small number of cracks with a large width is much more harmful for the material than the occurrence of a dense cracks network but with small width.

**Figure 9:**
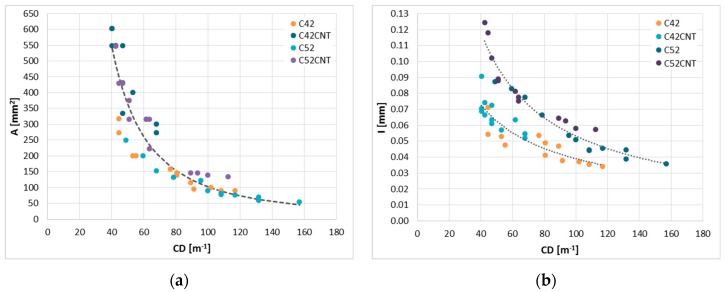
Relationships between parameters of the cracking patterns: (**a**) $\bar{A}\left(CD\right)\;$; (**b**) $\bar{I}\left(CD\right)$.

Figure 9 shows the relationships between the *CD* and the two cracking patterns parameters described earlier, i.e., $\bar{A}$ and $\bar{I}$. In the case of these relationships, the greatest degree of curve fit to the empirical data was obtained for the curve form adopting the power function equation. The global correlation coefficient between $\bar{A}$ and *CD* takes a high negative value of −0.83. With this in mind, the global functional curve equation was calculated, which with the diagnostic statistics is presented in Table 5. A very good degree of curve fitting to the empirical data was achieved (*R*^2^ = 0.86). Despite this fact, the relative estimation error is as much as 24.95%, which may cause inaccuracies in predicting the $\bar{A}$ values from *CD*. In the case of the $\bar{I}\left(CD\right)$, on the other hand, the global correlation coefficient was less favorable, equal to −0.69. Analyzing the distribution of points on the graph, it was noticed that the data can be described with good accuracy in two groups, making the division dependent on the class of cement used. It was not observed that the MWCNTs addition had a key impact on the description of the $\bar{I}\left(CD\right)$ relationship. A much better degree of curve fitting to the empirical data was obtained for samples made with CEM I 52.5R (*R^2^* = 0.94). In both cases, however, *W* assumes low values, which means that the $\bar{I}$ estimation based on *CD* will be subjected to a small error, maintaining the criterion of the class of cement used.

### 4.3. Correlations between the Properties of the Cracking Patterns and Physico-Mechanical Features of the Cement Matrix

Table 6 shows the correlation coefficients between the parameters describing the cracking patterns and the physico-mechanical properties of cement pastes. The absolute values of the correlation coefficients mostly indicate a strong correlation (|r|>0.7). The strongest correlations occur with the *CD*, dependencies with *f_c_*_(*R*)_, *f_c_*_(*T*)_, *f_cf_*_(*R*)_, and *S*_28(*R*)_ are even very strong (|r|>0.9), which positively affects the possibility of accurate estimation of these parameters based on *CD*. Similarly, in the case of $\bar{L}$, very strong correlations occur in five cases, i.e., with *f_c_*_(*R*)_, *D*_(*R*)_, *D*_(*T*)_, *S*_28(*R*)_, and *S*_28(*T*)_.

The $\bar{I}$ is the least correlated with physico-mechanical parameters. Here, in each case there is an average correlation (0.7>|r|>0.4). However, the weakest correlation is between *f_cf_*_(*T*)_ and the parameters of the cracking patterns. A similar observation was made in [47]. In the case of correlation with $\bar{A}$, $\bar{L},$ and *CD* it is weak (0.4>|r|). This situation makes it impossible to accurately estimate the tensile strength obtained after loading the samples with elevated temperature based on the characteristics of the cracking patterns.

The above knowledge of which parameters of the cracking patterns and with what accuracy can be used for the purposes of estimation and assessment of physico-mechanical parameters of cementitious material, is also of practical importance. In order to perform the estimation, it would be necessary to create functional equations that would make the values of the cracking patterns parameters dependent on the values of the physico-mechanical properties. This can be the basis for the development of new non-destructive testing methods for assessing the durability of a cementitious composite operating in a real construction.

## 5. Conclusions

The paper presents the results of research and analysis of the cracking patterns that were formed on the surface of the cement paste as a result of increased temperature loading. Based on the analyzed and interpreted research results, the final conclusions were formulated:

- In order to facilitate the image analysis process, the samples were subjected to the preparation, which consisted in applying the thin white acrylic film to the scanned surface. The statistical analyses carried out have shown that the preparation method applied does not affect the correctness of the results obtained from the image analysis.
- As the w/c ratio increased, the $\bar{A}$, $\bar{L}$, and $\bar{I}$ also increased, and *CD* decreased. Cement pastes made of cement with greater fineness (C52 and C52CNT) were characterized by smaller values of $\bar{A}$ and $\bar{L}$, higher values of *CD*, while in the case of $\bar{I}$ no clear influence of the cement class was observed. The presence of MWCNTs resulted in a significant increase in the $\bar{A}$, $\bar{L}$, and $\bar{I}$, and a decrease in *CD*.
- The use of MWCNTs in the form of an aqueous dispersion in the presence of a surfactant, which is SDS, resulted in foaming of the cement matrix during mechanical mixing. The result was a significant reduction in the cement matrix density and, thus, an increase in porosity. This is the main reason for the changes in the physico-mechanical properties of the cement matrix and it also had a key impact on the characteristics of the cracking patterns. The conclusions drawn from this work concern the combined effect of using MWCNTs and SDS, not the MWCNT addition itself.
- The relationship between the parameters of the cracking patterns was assessed. There is a very strong relationship between $\bar{A}$ and $\bar{L}$, and the global correlation coefficient between these parameters is up to 0.98. This relationship is constant and independent of technological variables of the material and confirms the fractal nature of the cracking patterns.
- The relationship between $\bar{A}$ and $\bar{I}$ largely depends on the series, i.e., mainly on the class of cement used and the presence of MWCNTs.
- The *CD* relationship was assessed with both $\bar{A}$ and $\bar{I}$. A global relation with a strong correlation was noted between *CD* and $\bar{A}$. However, in the case of the dependence between *CD* and $\bar{I}$, a clear effect of the cement class was found.
- The possibility of estimation of physico-mechanical properties of the cement paste based on the parameters of cracking patterns was evaluated. In the vast majority, the accuracy of the estimation would be at a very good level apart from the tensile strength estimation.

## Figures and Tables

**Table 1:** The results of the physico-mechanical characteristics of the cement pastes [50].

Series	C42			C42CNT			C52			C52CNT		
Water/Cement Ratio	0.4	0.5	0.6	0.4	0.5	0.6	0.4	0.5	0.6	0.4	0.5	0.6
*f_c_*_(*R*)_ (MPa)	61.40	43.43	34.17	26.70	19.02	15.22	69.52	49.67	39.10	36.49	27.87	22.62
*f_c_*_(*T*)_ (Mpa)	39.26	23.08	15.90	16.28	10.94	8.35	49.29	30.42	21.13	27.21	19.92	15.78
*f_cf_*_(*R*)_ (Mpa)	5.80	4.35	3.16	3.99	3.03	2.41	5.74	4.39	3.86	4.12	3.25	2.45
*f_cf_*_(*T*)_ (Mpa)	2.97	2.59	1.32	2.56	1.81	1.33	1.53	1.35	1.16	2.02	1.55	1.18
*D*_(*R*)_ (g/cm^3^)	1.692	1.528	1.416	1.143	1.062	0.952	1.750	1.574	1.432	1.182	1.110	0.995
*D*_(*T*)_ (g/cm^3^)	1.513	1.376	1.277	1.025	0.956	0.865	1.549	1.388	1.266	1.035	0.982	0.883
*S*_28(*R*)_ (mm/m)	2.25	2.52	3.23	3.56	3.77	4.06	1.69	2.44	3.13	3.17	3.75	4.40
*S*_28(*T*)_ (mm/m)	5.50	6.35	7.58	8.60	10.15	10.92	4.53	5.90	7.48	8.67	10.10	11.06

**Table 2:** The Shapiro-Wilk test results.

		C42—Before Preparation			C42—After Preparation		
w/c		0.4	0.5	0.6	0.4	0.5	0.6
Population size		260	174	107	248	182	101
The size of the random sample		248		101		174	
$\;\bar{A}\;$	*p*-value	2.34 × 10^−5^	7.17 × 10^−4^	1.44 × 10^−3^	2.10 × 10^−4^	4.02 × 10^−8^	3.35 × 10^−3^
	*p*-value *≤ α*	yes	yes	yes	yes	yes	yes
$\;\bar{L}\;$	*p*-value	5.14 × 10^−3^	2.87 × 10^−3^	0.02	8.74 × 10^−4^	3.72 × 10^−5^	0.07
	*p*-value *≤ α*	yes	yes	yes	yes	yes	no

**Table 3:** The Mann-Whitney *U* test results.

	C42—Before Preparation vs. C42—After Preparation					
Parameter	$\;\bar{A}\;$			$\;\bar{L}\;$		
w/c	0.4	0.5	0.6	0.4	0.5	0.6
*U*	26,102	12,548	4738	26,925	12,806	4801
*U_crit_* (two-sided test)	25,226	11,754	4235	25,574	12,101	4244
*U ≤ U_crit_*	no	no	no	no	no	no

**Table 4:** Equations of the functional curves describing the $\bar{A}\left(\bar{I}\right)$ relationship divided into series; *R^2^, S_e_, W*—diagnostic statistics; ρ—correlation coefficient.

Series	Equation	R^2^	*S_e_*(mm^2^)	*W* (%)	$\rho —\bar{A}/\bar{I}\;$	
C42	$\;\bar{A}$ $\;=\;53,570\;\cdot \;\bar{I}\;$ ^2^ $\;+\;808.28\;\cdot \;\bar{I}\;$	0.82	33.39	20.82	0.66	0.91
C42CNT	$\;\bar{A}$ $\;=\;8211\;\cdot \;\bar{I}\;$ ^2^ $\;+\;6319.65\;\cdot \;\bar{I}\;$	0.44	98.24	21.61		0.67
C52	$\;\bar{A}$ $\;=\;20,643\;\cdot \;\bar{I}\;$ ^2^ $\;+\;762.54\;\cdot \;\bar{I}\;$	0.94	15.28	13.42		0.96
C52CNT	$\;\bar{A}$ $\;=\;23,050\;\cdot \;\bar{I}\;$ ^2^ $\;+\;1492.60\;\cdot \;\bar{I}\;$	0.90	45.96	15.68		0.96

**Table 5:** Equations of the functional curves describing the $\bar{A}\left(CD\right)$ and $\bar{I}\left(CD\right)$ relationship; R^2^, S_e_, W—diagnostic statistics; ρ—correlation coefficient.

Series	Equation	R^2^	S_e_	*W* (%)	$\rho –\bar{A}/\bar{I}\;$	
$\;\bar{A}\left(CD\right)\;$						
All	$\;\bar{A}$ = 470,138 · *CD*^−1.831^	0.86	63.74 mm^2^	24.95	−0.83	
$\;\bar{I}\left(CD\right)\;$						
C42 + C42CNT	$\;\bar{I}$ = 0.901 · *CD*^−0.682^	0.78	0.0067 mm	12.20	−0.69	−0.86
C52 + C52CNT	$\;\bar{I}$ = 2.997 · *CD*^−0.875^	0.94	0.0060 mm	8.63		−0.92

**Table 6:** Correlation matrix of the cement pastes parameters.

Parameter	*f_c_*_(*R*)_	*f_c_*_(*T*)_	*f_cf_*_(*R*)_	*f_cf_*_(*T*)_	*D*_(*R*)_	*D*_(*T*)_	*s*_28(*R*)_	*s*_28(*T*)_
$\;\bar{A}\;$	−0.89	−0.83	−0.86	−0.32	−0.88	−0.86	0.88	0.90
$\;\bar{L}\;$	−0.94	−0.87	−0.88	−0.28	−0.93	−0.91	0.94	0.94
$\;\bar{I}\;$	−0.71	−0.62	−0.79	−0.57	−0.76	−0.78	0.85	0.82
*CD*	0.92	0.95	0.91	0.31	0.80	0.78	−0.90	−0.84

## Appendix A

### Appendix A.1. The $\bar{A}$ and $\bar{L}$ Measurement Procedure Using the ImageJ v. 1.51j8 Software

- Opening the scan (*File > Open*).
- Setting the image scale (*Analyze > Set Scale*)—in order to correctly determine the surface areas and perimeter of clusters, the value of the scan resolution should be introduced.
- Rotating the image to position the sample longitudinal axis horizontally on the screen (*Image > Transform > Rotate*)—it may happen that the sample was not placed on the scanner parallel to the scanning traverse, but at a small angle—this facilitates further work with the image.
- Selection of the area that will be measured with dimensions of 157 × 38.5 mm on the sample surface (*Edit > Selection > Specify*)—this resulted from the necessity to determine a constant surface area for each sample because as a result of a sudden increase in temperature, the cement paste was subjected to the volume deformations.
- Cropping the image to 157 × 38.5 mm (*Image > Crop*).
- Contrasting the image (*Image > Adjust > Brighthness/Contrast* or *Image > Adjust > Window/Level*)—in order to “display” graphically the lines forming cracks in relation to the cluster surface.
- Creation a mask from a contrasted image (*Image > Overlay > Add Image*), then adding the mask to the *ROI Manager* (*Image > Overlay > To ROI Manager*)—the mask at a later stage can be used as a foundation with a given clearance (in the *ROI Manager*: *Properties > Opacity*),
- Creation a binary image (*Process > Binary > Make binary*)—a black-and-white image of the sample surface is created. The cracks are then visible as black (0 on the histogram), and the surface of the clusters is visible as white (255 on the histogram).
- Removal of any discontinuities in the lines forming the cracks (from the toolbar: *Brush* with a size equal to 2 px, with a gradient value corresponding to the crack gradient, 0)—to facilitate the work, a primer from a previously created mask can be applied (in the *ROI Manager*: select *Show All*).
- Removal of noise generated on the cluster surface (from the toolbar: *Brush* of any size, with a gradient value corresponding to the cluster surface gradient—255).
- Selection of the quantities to be measured—that is, the area and the perimeter of the cluster (*Analyze > Set Measurements >* selection of: *Area, Perimeter*).
- Inversion of colors in the image (*Edit > Invert*)—this is required in order to correctly identify the cluster surfaces by the counting module, which only analyzes areas with a gradient value equal to 0 (the object tested is black, the background is white).
- Automatic measurement of the surface area and perimeter of each cluster present on the sample surface using the *Analyze Particles* counting module (*Analyze > Analyze Particles*)—in the module settings must be defined: *size (mm^2^)—2—infinity*, *show: Overlay* (an image is created with the identification numbers that have been assigned to each of the clusters). The cluster surface area and perimeter along with its identification number are exported to a text file—for this purpose, in the module settings, the *display results* option should be additionally selected.
- Calculation of average values from the $\bar{A}$ and $\bar{L}$ measurements, e.g., using Microsoft Excel spreadsheet software.

### Appendix A.2. The $\bar{I}$ Measurement Procedure Using the ImageJ v. 1.51j8 Software

- Opening the scan (*File > Open*).
- Setting the image scale (*Analyze > Set Scale*)—in order to correctly determine the width of the cracks, the value of the scan resolution should be introduced.
- Rotating the image to position the sample longitudinal axis horizontally on the screen (*Image > Transform > Rotate*)—it may happen that the sample was not placed on the scanner parallel to the scanning traverse, but at a small angle. This facilitates further work with the image.
- Creating a parallel line to the longitudinal axis of the specimen, in a half the beam height (from the toolbar: *Straight line*—stake out with the *Shift* key pressed creates a vertical or horizontal line).
- Using the image magnification, check that the line created does not cut any cracks with an angle of inclination less than about 45°. However, if this situation occurs, the vertical position of the line should be corrected, but in such a way that the line is not more than 5 mm from the top or bottom of the longitudinal axis of the specimen.
- Removal of noise between cracks along the line length (from the toolbar: *Brush*)—it may happen that in the place where the line runs, there is a discoloration on the surface of the sample, which can be mistakenly interpreted as a crack at the stage of further analysis.
- Creating a graph showing the gradient value of pixels located on the line as a function of its length (*Analyze > Plot Profile*)—the gradient value is the same as the image mode obtained by scanning (in this case it is a monochrome image—8-bit gray scale). By default, the line has a thickness of 1 px.
- Counting how many cracks are cut by the line—pixels with a gradient value less than 200 are considered as cracks.
- Exporting numerical data from the graph to a text file (in the graph pane: *List*).
- Counting the number of pixels whose gradient value is below 200 importing a text file to the Microsoft Excel spreadsheet and applying the conditional function (*Countif*).
- Calculation of $\bar{I}$ as the quotient of the number of pixels interpreted as cracks to their total number. The average crack width can be represented in the unit (px/crack) or (mm/crack).

### Appendix A.3. The CD Measurement Procedure Using the ImageJ v. 1.51j8 Software

- Opening the scan (*File > Open*).
- Setting the image scale (*Analyze > Set Scale*).
- Rotating the image to position the sample longitudinal axis horizontally on the screen (*Image > Transform > Rotate*)—it may happen that the sample was not placed on the scanner parallel to the scanning traverse, but at a small angle. This facilitates further work with the image.
- Selection of the area that will be measured with dimensions of 157 × 38.5 mm on the sample surface (*Edit > Selection > Specify*)—this resulted from the necessity to determine a constant surface area for each sample because as a result of a sudden increase in temperature, the cement paste was subjected to the volume deformations.
- Cropping the image to 157 × 38.5 mm (*Image > Crop*).
- To create three test lines parallel to the longitudinal axis of the beam and dividing the sample surface into four equal parts, use the *Grid Overlay* macro should be used (*Plugins > Compile and Run*).
- After running the *Grid Overlay* macro in the dialog box, the tiles sizes should be specified. To divide the sample surface into 4 equal parts using horizontal test lines, enter the total surface length in pixels in the *Tile width* field, and enter the value of the scan surface height divided by four in the *Tile height* field.
- Counting how many cracks are cut by each test line on the sample surface.
- Using, e.g., Microsoft Excel spreadsheet software, calculate the average number of cracks that occur over the length of test lines for a given series of samples and the w/c ratio.
- Calculation of *CD* as the quotient of the average number of cracks occurring on the test line to the length of the scan expressed in meters, i.e., 0.157 m.
